# Sterile Reverse Osmosis Water Combined with Friction Are Optimal for Channel and Lever Cavity Sample Collection of Flexible Duodenoscopes

**DOI:** 10.3389/fmed.2017.00191

**Published:** 2017-11-07

**Authors:** Michelle J. Alfa, Harminder Singh, Zoann Nugent, Donald Duerksen, Gale Schultz, Carol Reidy, Patricia DeGagne, Nancy Olson

**Affiliations:** ^1^St. Boniface Research Centre, Winnipeg, MB, Canada; ^2^Department of Medical Microbiology, University of Manitoba, Winnipeg, MB, Canada; ^3^Department of Internal Medicine, University of Manitoba, Winnipeg, MB, Canada; ^4^Winnipeg Regional Health Authority, Winnipeg, MB, Canada; ^5^St. Boniface Hospital, Winnipeg, MB, Canada

**Keywords:** lever cavity, biofilm, channel, PTFE-BBF model, endoscope sample collection, duodenoscope, flush-brush-flush extraction

## Abstract

**Introduction:**

Simulated-use buildup biofilm (BBF) model was used to assess various extraction fluids and friction methods to determine the optimal sample collection method for polytetrafluorethylene channels. In addition, simulated-use testing was performed for the channel and lever cavity of duodenoscopes.

**Materials and methods:**

BBF was formed in polytetrafluorethylene channels using *Enterococcus faecalis, Escherichia coli*, and *Pseudomonas aeruginosa*. Sterile reverse osmosis (RO) water, and phosphate-buffered saline with and without Tween80 as well as two neutralizing broths (Letheen and Dey–Engley) were each assessed with and without friction. Neutralizer was added immediately after sample collection and samples concentrated using centrifugation. Simulated-use testing was done using TJF-Q180V and JF-140F Olympus duodenoscopes.

**Results:**

Despite variability in the bacterial CFU in the BBF model, none of the extraction fluids tested were significantly better than RO. Borescope examination showed far less residual material when friction was part of the extraction protocol. The RO for flush-brush-flush (FBF) extraction provided significantly better recovery of *E. coli* (*p* = 0.02) from duodenoscope lever cavities compared to the CDC flush method.

**Discussion and conclusion:**

We recommend RO with friction for FBF extraction of the channel and lever cavity of duodenoscopes. Neutralizer and sample concentration optimize recovery of viable bacteria on culture.

## Introduction

The recent outbreaks due to contaminated flexible endoscopes (1, 2) have raised questions regarding the frequency and optimal method to use for sampling endoscopes especially duodenoscopes (2–11). Endoscope culture results may be affected by various factors including viable but non-culturable (VBNC) organisms (5, 12, 13), use of neutralizer to ensure protection and growth of damaged organisms (5, 10, 12, 14, 15), type of fluid used to extract samples from channels (3, 16), and type of friction [e.g., brush or swab for extraction from channels and lever cavity (4, 5, 11, 16)]. Indeed, Kim and Muthusamy (6) stated: *“*… *a negative culture does not ensure sterility or even exclude the possibility of a contaminated duodenoscope*.” Biofilm developing and accumulating in patient-used endoscopes with repeated rounds of reprocessing (10, 17) has been recognized as an issue associated with moisture in channels during storage (18). Borescope examination of patient-used endoscopes has shown visible water droplets in 95% of endoscope suction channels (19). Furthermore, the development of biofilm within the channels and lever cavity of duodenoscopes and fixation of biofilm during high-level disinfection (HLD) have been identified as additional challenges to sample collection (4, 10). The biofilm model using PTFE channels has been recommended as appropriate for evaluating endoscope cleaning (ISO 15883-5). However, this PTFE-biofilm model does not incorporate the fixation step that occurs when disinfectants are used as part of endoscope reprocessing. Indeed, the recent buildup biofilm (BBF) model in polytetrafluorethylene channels (PTFE-BBF) that mimics repeated rounds of biofilm formation and fixation by glutaraldehyde is the first to mimic the in-use conditions that challenge sample collection (13). There have been few published reports using any of the published model systems that compare different channel extraction fluids, and type of friction used to improve sample recovery.

The aim of this study was to utilize the PTFE-BBF model to evaluate various extraction fluids and the role of friction in sample collection efficacy and then evaluate the optimal method using simulated-use testing in duodenoscopes.

## Materials and Methods

### Bacteria Used for Suspension Testing and for BBF Formation

The bacteria used included *Escherichia coli* ATCC 25922, *Pseudomonas aeruginosa* ATCC 15442, and *Enterococcus faecalis* ATCC 29212. The bacterial stocks of these organisms were stored at −70°C and prior to use in experiments they were subcultured three successive times onto blood agar (BA) media consisting of tryptone soya agar containing 5% (v/v) whole sheep blood (Oxoid, Nepean, ON, Canada). For all experimental testing, the bacterial cultures used were 24 h old.

### Effect of Extraction Fluid on Viability of Bacteria

*Enterococcus faecalis* and *P. aeruginosa* were suspended in each of the extraction fluids to be tested (Table 1) to a concentration of approximately 4 Log_10_ CFU/mL. Viable counts were determined at time 0 and compared to the viable counts after the extraction fluid suspensions were held at 2 and 24 h at room temperature. The viable count was performed using serial 1:10 dilutions of the test suspension and plating 0.1 mL of the direct sample and each dilution onto BA plates. The plates were incubated for 24 h and the CFU/mL determined.

**Table 1:** Summary of extraction fluids and brushes evaluated.

Sterile fluid used	Flush^a^	Flush-brush-flush^a^	Flush-pull through-Flush^a^
RO water	*Sample*: 40 mL RO water flushed through channel	*Sample*: – 20 mL RO water flushed through channel – bristle brush passed through once then head cutoff into sample – 20 mL RO water flush	*Sample*: – 20 mL RO water flushed through channel – pull-through passed through once then head cutoff into sample – 20 mL RO water flush

	*Neutralizer*: 40 mL added to sample collection container	*Neutralizer*: 40 mL added to sample collection container	*Neutralizer*: 40 mL added to sample collection container

RO water + 0.02% Tween80 (Tween 80; Sigma, St Louis, MO, USA)	*Sample*: 40 mL RO + Tween flushed through channel	*Sample*: – 20 mL RO + Tween flushed through channel – bristle brush passed through once then head cutoff into sample – 20 mL RO + Tween flushed through channel	*Not applicable*

	*Neutralizer*: 40 mL added to sample collection container	*Neutralizer*: 40 mL added to sample collection container	

Phosphate-buffered saline (PBS)	*Sample*: 40 mL PBS flushed through channel	*Sample*: – 20 mL PBS flushed through channel – bristle brush passed through once then head cutoff into sample – 20 mL PBS flushed through channel	*Not applicable*

	*Neutralizer*: 40 mL added to sample collection container	*Neutralizer*: 40 mL added to sample collection container	

PBS + 0.02% Tween80	*Sample*: 40 mL PBS flushed through channel	*Sample*: – 20 mL PBS + Tween flushed through channel – bristle brush passed through once then head cutoff into sample – 20 mL PBS + Tween flushed through channel	*Not applicable*

	*Neutralizer*: 40 mL added to sample collection container	*Neutralizer*: 40 mL added to sample collection container	

Dey–Engley broth^b^ (BD Difco, Canada)	*Sample*: 40 mL DE broth flushed through channel 40 mL DE broth added to sample collection container	*Sample*: – 20 mL DE flushed through channel – bristle brush passed through once then head cutoff into sample – 20 mL DE flushed through channel – 40 mL DE added to sample collection container	*Not applicable*

Letheen broth^b^ (Remel, Lenexa KS, USA)	*Sample*: 40 mL L broth flushed through channel 40 mL L broth added to sample collection container	*Sample*: – 20 mL L broth flushed through channel – bristle brush passed through once then head cutoff into sample – 20 mL L broth flushed through channel – 40 mL L broth added to sample collection container	*Not applicable*

CDC channel sample method	*Sample*: 50 mL RO water flushed through channel	*Not applicable*	*Not applicable*
	*Neutralizer*: None added		

*^a^Total volume of sample collected from each channel tested was 40 mL. The total volume of sample after neutralizer was added was 80 mL. The channel friction methods evaluated included a commercial bristle cleaning brush or a commercial pull-through channel cleaning device*.

*^b^No additional neutralizer needed for DE and LB as they are neutralizing broths. To keep total volume of all samples the same (i.e., 80 mL), there was 40 mL of Dey–Engley broth or Letheen broth added to these channel samples to give a total sample volume of 80 mL*.

### PTFE-BBF Model Used for Testing Channel Sample Collection Methods

As described by Alfa et al. (20, 21) BBF was formed over eight days at room temperature inside PTFE channels (Endoscopy Development Company, Maryland Heights, MO, USA) using ATS-2015 (Healthmark, Fraser, MI, USA) containing 8 Log_10_/mL (day 1) of *E. faecalis* and *P. aeruginosa*. On days 3, 4, and 5, the PTFE channels were rinsed and exposed to glutaraldehyde partial fixation (1:50 dilution of glutaraldehyde) and then repeat biofilm formation allowed to develop overnight (13). Once the BBF was fully formed on day 8, there was full HLD of the BBF using 2.6% glutaraldehyde (Metricide^®^ from Metrex—Sybron Canada, Oakville, ON, Canada) for 20 min at room temperature. Segments (5 cm) of the fully formed BBF were cut from the full PTFE-BBF channel and attached in between two 60 cm sterile PTFE segments to form a “surrogate endoscope channel” (SEC) that was 125 cm long as described by Alfa et al. (20, 21). The SEC model was used to mimic low levels of organisms within the BBF that was only present in the central 5 cm portion of the total instrument channel length. The SEC was used to assess the various channel extraction methods.

### Methods Assessed for Sample Collection for PTFE-BBF Channels

The PTFE-BBF segments were assessed using flush only as well as flush-brush-flush (FBF) collection methods. Table 1 summarizes the various extraction fluids and channel friction devices evaluated in this study. All testing was done using five replicates. The CFU for positive controls was determined using destructive testing of samples where each 5 cm PTFE segment was aseptically cut length-wise and cross-wise into 10 small pieces (each about 1 cm × 0.5 cm) and all pieces were totally immersed in 5 mL of neutralizer. For the test samples, neutralizer (9) was added at a 1:1 ratio to each extracted sample and then samples were subjected to sonication for 5 min followed by vortex mixing for 1 min. Each sample was 80 mL (Table 1). All samples had direct counts performed by inoculating a BA plate with 0.1 mL of the original sample (fluid spread over the surface of the agar). Concentration was done by centrifugation of 35 mL of sample and all but 0.4 mL of the supernatant was removed and the pellet was resuspended in this fluid and then the total sample was inoculated and spread over the surface of a second BA plate. Concentration was also done by filtration of 35 mL of sample through a 0.45-μm Nalgene grid filter unit. The filter was aseptically removed and transferred to a BA plate. The inoculated BA plates were incubated at 35°C for 72 h and colonies counted. Results were presented as CFU/5 cm segment.

### Endoscope Inoculation and Extraction Methods

#### Endoscope Lever Cavity Inoculation and Extraction Methods

To assess the optimal method of obtaining a sample from the duodenoscope lever cavity the same inoculation method was used as described by Alfa et al. (20, 21). Briefly, ATS-2015 containing approximately 10^5^ CFU/mL of both *E. faecalis* and *E. coli* was used to inoculate the lever cavity of both a JF-140F duodenoscope and a TJF-Q 180V duodenoscope with 0.1 mL of inoculum (total inoculum in lever cavity was 10^4^ CFU). The lever mechanism was articulated up-down three times to ensure the inoculum was thoroughly spread and then the lever was left in the vertical position and the inoculum allowed to dry for 2 h.

For extraction from the lever cavity, a FBF method described by Alfa et al. (20, 21) was used and compared with recently recommended CDC method (16). The FBF method consisted of 1.0 mL of sterile reverse osmosis (RO) water instilled into the lever cavity (lever in raised vertical position) using a sterile plastic transfer pipette (Fisherbrand, Ottawa, ON, Canada), and the fluid was allowed to dwell in the cavity for 1 min. The lever was adjusted to the mid-way position and a sterile cleaning brush (MAJ-1888) (Olympus Inc., Center Valley, PA, USA) was then used to scrub both sides of the lever and the cavity. The head of the brush was cutoff into the sample collection container using sterile scissors. The remaining cavity fluid was flushed up-down a total of five times and then all the fluid was transferred into the same sterile collection container containing the brush head. An additional 1 mL of RO water was transferred into the lever cavity and the lever articulated up-down three times. The cavity fluid was flushed up-down five times and then transferred into the sample collection container. An equal volume of neutralizer (9) was added to the sample (total volume of cavity and neutralizer was 4 mL). Because the inoculum was high enough (i.e., 10^4^ CFU/cavity) no concentration method was used for either the CDC or FBF collection methods. Each sample was sonicated for 5 min and vortex mixed for 1 min and then the sample was serially diluted 1:10 and then 100 μL of the direct sample and each dilution was inoculated and spread over the surface of a BA plate and incubated for 72 h.

For the CDC method (16), the lever cavity sample was serially diluted 1:10 and 100 μL of the direct sample and each dilution were inoculated and spread over the surface of separate BA plates. Results were calculated as percentage of inoculum recovered.

#### Endoscope Channel Inoculation and Extraction Methods

The suction channel was inoculated with ATS-2015 containing *E. faecalis* and *E. coli* at 10^5^ CFU/mL by instilling 1 mL of the inoculum into the distal end of the suction channel and elevating the distal end so the inoculum ran down the channel toward the instrument port (total inoculum per channel was 10^5^ CFU). Air was suctioned through the instrument port for 10 min and then the inoculated duodenoscope was dried at room temperature for 2 h. Results were presented as percentage of inoculum recovered.

**Figure 1:**
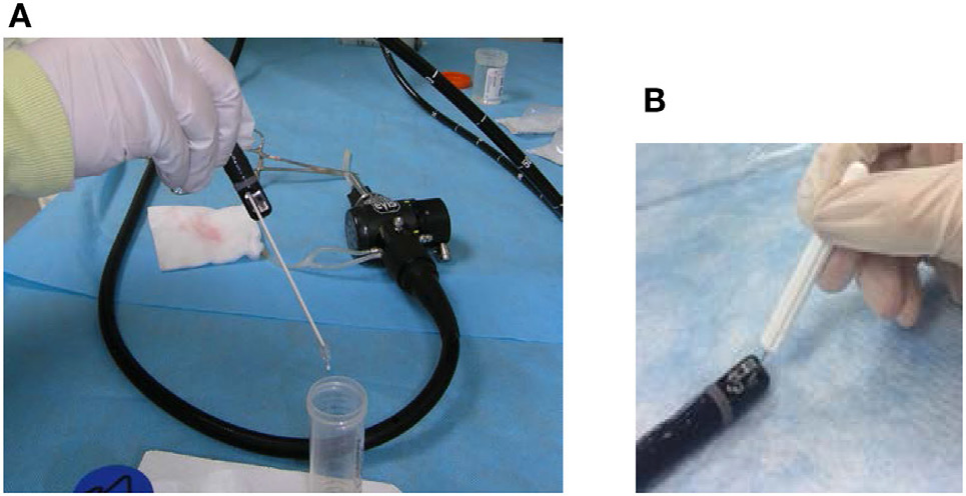
Bristle brushes used for sample collection. The brushes used included an appropriately sized bristle brush for the instrument channel for the flush-brush-flush (FBF) sample collection **(A)** and a tiny bristle brush for FBF lever cavity sample collection **(B)**.

The duodenoscope channel extraction was done as described by the CDC (16) protocol as well as by our FBF protocol. Our FBF protocol is outlined in Table 1. It included friction (sterile tiny cavity bristle brush, Olympus part MAJ-188) as well as addition of neutralizer to the final sample. Figure 1 shows the brush used for the channel sampling as well as the tiny bristle brush used for the lever cavity sampling. Because the inoculum counts were high enough (i.e., 10^5^ CFU), no concentration methods were used for either the CDC or FBF protocol. The direct sample collected was serially diluted 1:10 and 100 μL of the direct sample and each dilution were inoculated and spread over the surface of separate BA plates and incubated for 72 h. Results were calculated as percentage of inoculum recovered.

### Statistical Analysis

Descriptive analysis was performed to describe the data. To assess the impact of extraction fluids on viability of *E. faecalis* and *P. aeruginosa* the paired *t*-test was used to compare baseline inoculum counts and counts at 2 and 24 h as well as between the counts at 2 and 24 h. To determine the effect of different extraction fluids and friction methods on bacterial recovery from the PTFE-BBF model, the counts from each extraction method were compared with controls and with each other using Kruskal–Wallis and Wilcoxon rank sum tests.

## Results

To determine the impact of the extraction fluids on viability *E. faecalis* and *P. aeruginosa* were suspended in the extraction fluids to be evaluated and viable count determined at time of inoculation and after holding for 2 and 24 h at room temperature (Table 2). Overall, *P. aeruginosa* and *E. faecalis* CFU were minimally impacted at 2 h for any of the extraction fluids tested (although the reduction of CFU was statistically significantly decreased for *P. aeruginosa* in RO and RO with 0.02% Tween80 this reduction was only about 0.5 Log_10_). There was a greater negative impact (reduction of CFU by 1–2 Log_10_) at 24 h for *P. aeruginosa* in RO water and PBS with or without 0.02% Tween80. There was significant replication of *E. faecalis* at 2 h (1 Log_10_ increase) and of both organisms when held for 24 h at room temperature in Dey–Engley broth or Letheen broth (3–5 Log_10_ increase in CFU by 24 h).

**Table 2:** Impact of extraction fluids on survival of *Enterococcus faecalis* and *Pseudomonas aeruginosa* at room temperature.

	*E. faecalis*					*P. aeruginosa*				
	Mean Log_10_ CFU/mL (SD)					Mean Log_10_ CFU/mL (SD)				
Extraction fluid	2 h	Inoculum versus 2 h	24 h	Inoculum versus 2 h	2 versus 24 h	2 h	Inoculum versus 2 h	24 h	*p* Value*	2 versus 24 h
			
		*p* Value*		*p* Value*	*p* Value**		*p* Value*			*p* Value**
RO water	4.615 (0.076)	0.31	4.769 (0.078)	0.11	0.13	4.017 (0.011)	0.03	3.234 (0.025)	0.006	0.0003
RO + 0.02% Tween80	4.806 (0.018)	0.03	4.829 (0.10)	0.10	0.76	4.029 (0.051)	0.03	2.140 (0.584)	0.03	0.03
Phosphate-buffered saline (PBS)	4.660 (0.011)	0.09	4.485 (0.091)	0.94	0.08	4.149 (0.018)	0.08	3.646 (0.021)	0.01	0.0001
PBS + 0.02% Tween80	4.748 (0)	0.047	4.811 (0.115)	0.12	0.44	4.636 (0.041)	0.32	3.646 (0.038)	0.01	0.002
Dey–Engley broth	5.087 (0.020)	0.005	9.401 (0.057)	0.0003	0.0001	4.713 (0.067)	0.22	7.408 (0.039)	0.004	0.003
Letheen broth	5.115 (0.084)	0.01	9.071 (0.031)	0.0003	0.0002	4.513 (0.034)	0.97	7.646 (0.078)	0.002	0.0002

*E. faecalis and P. aeruginosa were suspended in various extraction fluids (inoculum was; 4.49 ± 0.10 Log_10_ CFU/mL for E. faecalis and 4.52 ± 0.19 Log_10_ CFU/mL for P. aeruginosa). Survival of bacteria in suspension was assessed after holding for 2 or 24 h at room temperature. The results represent the average of three replicates. No neutralizer was added to any of the test samples*.

*These p values refer to the comparison with the inoculum.

**These p values refer to comparison between CFU at 2 and 24 h.

**Figure 2:**
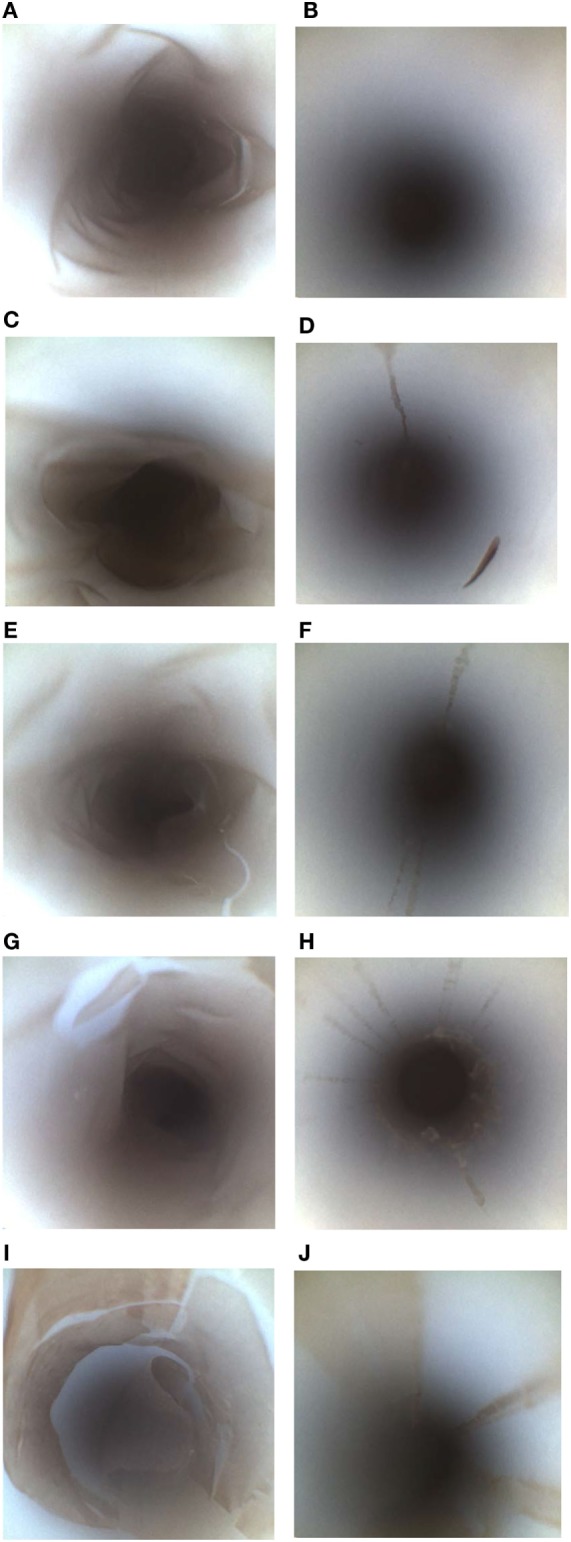

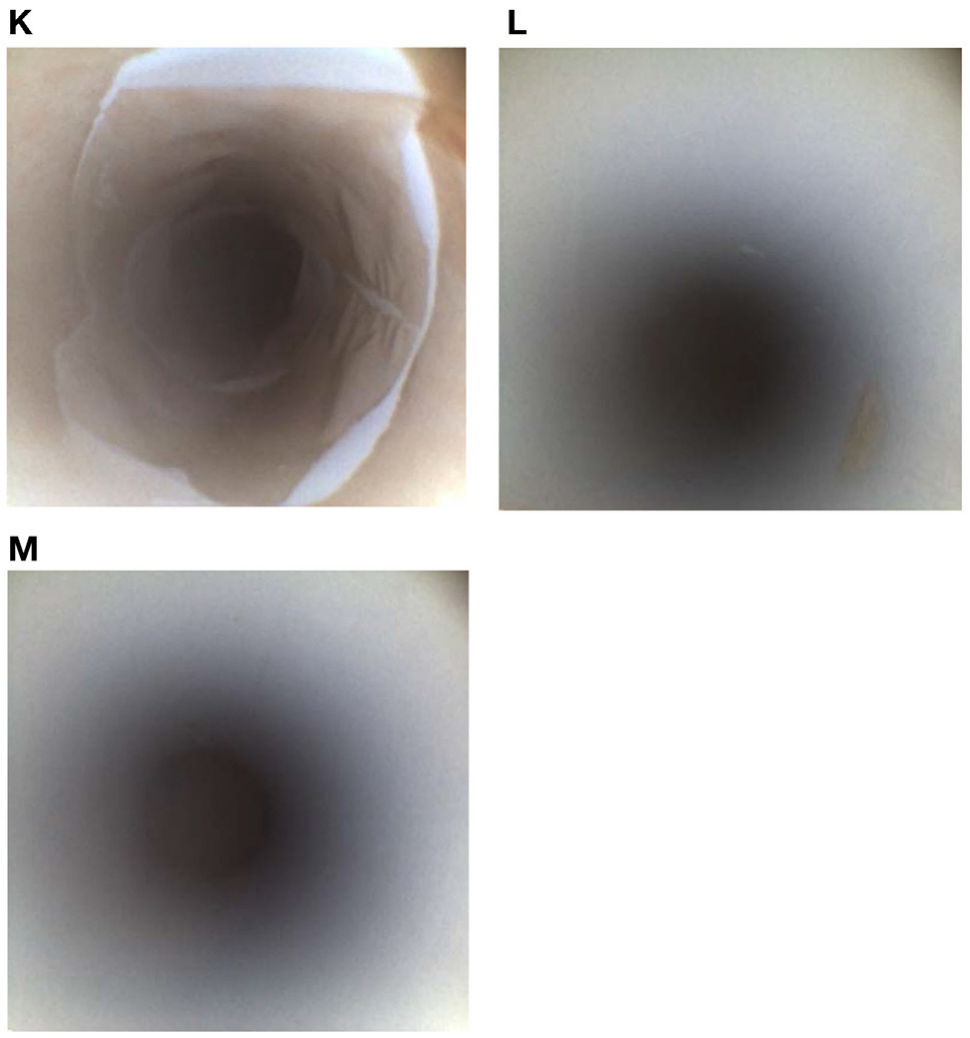
Borescope evaluation of various PTFE-BBF channel extraction methods. The PTFE-BBF surrogate endoscope channel was extracted using different methods. The positive control **(A)** and negative control **(B)** for the PTFE-BBF testing are shown. The various channel extraction methods tested included; RO Flush **(C)**, RO flush-brush-flush (FBF) **(D)**, phosphate-buffered saline (PBS)-Tween80 Flush **(E)**, PBS Tween 80 FBF **(F)**, RO-Tween80 Flush **(G)**, RO-Tween 80 FBF **(H)**, DE broth Flush **(I)**, DE broth FBF **(J)**, LB broth Flush **(K)**, LB broth FBF **(L)**, RO with pull-through channel cleaner **(M)**.

All the extraction fluids and friction methods listed in Table 1 were evaluated to determine the optimal extraction conditions for endoscope channels (Table 2). Among the different extraction fluids used as a flush only method, there was greater extraction of *E. faecalis* with RO than all other fluids flushed through the channel (*p* = 0.046). It is clear that there was variability in the viable counts for the various extraction conditions (Table 3) but the borescope examination (Figure 2) showed that all methods that incorporated friction left far less residual material inside the PTFE-BBF channel post-sample collection. The pull-through channel cleaner was the most effective at removing fixed residuals in the borescope examination.

**Table 3:** Comparison of sample extraction fluids with and without friction using the PTFE-BBF channel model.

Extraction conditions	*Enterococcus faecalis*	*Pseudomonas aeruginosa*

	Mean Log_10_ CFU/segment^b^ (SD)	Mean Log_10_ CFU/segment^b^ (SD)
**Positive control 1^a^**	0.67 (1.16)	<LD
RO flush	2.08 (1.28)*	1.76 (1.63)
RO + flush-brush-flush (FBF)	1.52 (1.97)	1.18 (1.52)
RO-Tween flush	<LD	<LD
RO-Tween + FBF	1.13 (1.95)	<LD
**Positive control 2^a^**	<LD	<LD
PBS flush	0.22 (0.38)	<LD
PBS + FBF	<LD	0.22 (0.38)
PBS-Tween flush	<LD	0.33 (0.33)
PBS-Tween + FBF	<LD	<LD
**Positive control 3^a^**	0.87 (1.50)	1.93 (1.90)
Dey–Engley broth flush	0.33 (0.32)	0.33 (0.32)
Dey–Engley + FBF	0.49 (0.58)	0.31 (0.54)
Letheen broth flush	2.25 (0.60)	1.18 (0.96)
Letheen broth + FBF	1.66 (1.33)	0.55 (0.48)
**Positive control 4^a^**	1.96 (0.45)	0.57 (0.98)
RO + Flush-Pull-through-Flush	1.65 (0.05)	1.23 (0.39)

*^a^The CFU for positive controls was determined using destructive testing of triplicate samples as described in the Section “Materials and Methods.” LD for positive controls that were cultured using destructive testing was 1 CFU/0.1 mL = 10 CFU/mL (50 CFU/segment)*.

*^b^All counts for sample extraction tests represent the mean of five replicate PTFE-BBF segments where each sample had neutralizer added and was concentrated by centrifugation. LD for concentrated test samples was 1 CFU/segment*.

*With RO flush, the extraction was significantly higher for *E. faecalis* (*p* = 0.046) than for the combination of all other flush only methods. There were no other significant differences.

Although the pull-through channel cleaner was the most effective by borescope examination, there was more aerosolization of the sample when the pull-through device exited the distal end of the suction channel compared to the bristle brush. The pull-through device had more sample loss and also created an increased workplace safety issue to staff collecting the sample so the bristle brush was selected as the optimal overall method for friction during endoscope channel sample extraction.

**Figure 3:**
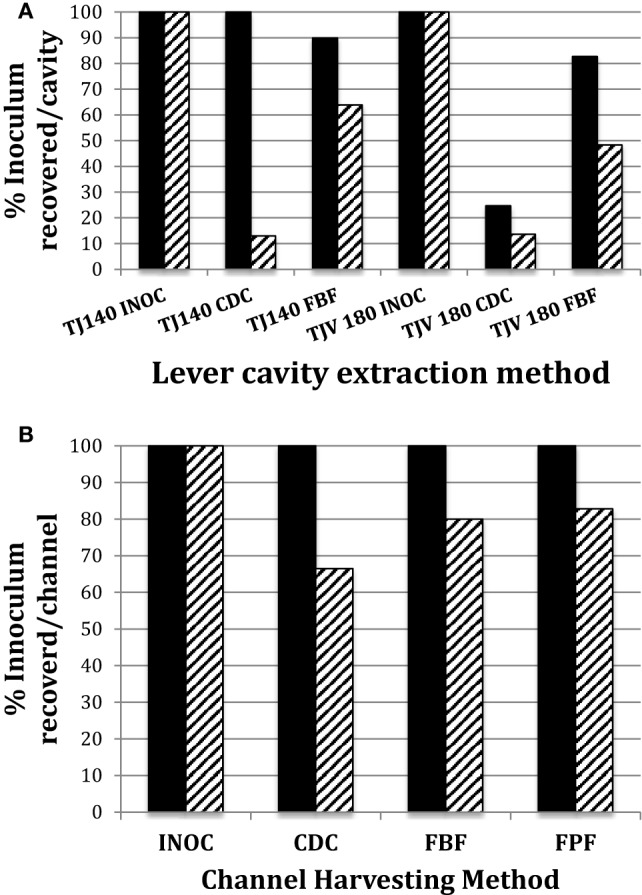
Comparison of CDC and flush-brush-flush (FBF) lever cavity sample collection from JF-140F and TJF-Q180V duodenoscopes. The endoscope suction channel from the instrument port to the distal end and the lever cavity were inoculated and dried as described in the Section “Materials and Methods.” The CDC cavity extraction method versus the FBF method for JF-140F (legend label; TJ140) and TJF-Q180 (legend label; TJV 180) lever cavities is shown in **(A)** and the CDC channel extraction method versus the FBF and Flush-Pull-through-Flush methods for the JF-140F channel are shown in **(B)**. Solid and hatched bars represent *Enterococcus faecalis* and *Escherichia coli*, respectively. The JF-140F inoculum/lever cavity **(A)** was Log_10_ 4.66 CFU and Log_10_ 3.55 CFU for *E. faecalis* and *E. coli*, respectively and Log_10_ 4.78 CFU and Log_10_ 4.52 CFU for *E. faecalis* and *E. coli* in the TJF-Q180V duodenoscope, respectively. The JF-140F inoculum/channel **(B)** was Log_10_ 5.56 CFU and Log_10_ 5.43 CFU for *E. faecalis* and *E. coli*, respectively. There was no statistically significant difference in the recovery of *E. faecalis* or *E. coli* for any of the channel extraction methods. However, using the combined data from all duodenoscopes tested, there was a statistically significant increase in the recovery of *E. coli* for the FBF cavity extraction method versus the CDC method (*p* = 0.017).

Thus, the optimal channel sampling method identified using the PTFE-BBF model consisted of RO as the extraction fluid combined with brushing (bristle brush) and flushing of the channel. The FBF extraction protocol was used for simulated-use testing of the instrument channel and level cavity (tiny bristle brush) of duodenoscopes and compared to the CDC sampling method (Figure 3). The FBF extraction protocol using RO provided significantly better recovery of *E. coli* (*p* = 0.02) from the duodenoscope lever cavity (both 140/160 and 180 duodenoscopes) compared to the CDC flush method. The difference for extraction of samples from duodenoscope channels (both 140/16 and 180 duodenoscopes) was not significant. There was no difference in *E. faecalis* extraction either from the lever cavity or channel between the FBF extraction protocol and the CDC flush method.

## Discussion

The key issues in sample collection methods used to assess contamination of flexible endoscopes includes protection of organism viability and CFU count during transport of the sample, extraction efficiency of both Gram-positive, Gram-negative, and fungal organisms from fixed residuals within the endoscope channel, ensuring stimulation of growth for VBNC bacteria that may be present in the sample, compatibility with the endoscope materials and concentration of the sample to improve the limit of detection on culture. Although there have been a number of studies published using many of the extraction fluids that we tested (3, 4, 9–11, 18), our data are the first to show that the extraction fluids can detrimentally affect the viability of the bacteria held for 24 h at room temperature. There was a 1–2 Log_10_ reduction in viable *E. coli* but not *E. faecalis* in RO and PBS with or without 0.02% Tween80 over a 24-h period. This suggests if endoscope samples were collected with these extraction fluids and transported at room temperature overnight, there could be a detrimental effect on the viable count for Gram-negative bacteria. Furthermore, our data showed that endoscope samples collected in Letheen or Dey–Engley broth held at room temperature showed a slight increase in CFU by 2 h and about a 3–4 Log_10_ increase in both *E. faecalis* and *E. coli* by 24 h. These data indicate that if samples are in broth media that they should be held on ice (or refrigerated) to prevent bacterial replication otherwise the CFU detected may lead to unnecessary action being taken. Refrigeration of clinical samples such as urine that require quantification is the accepted method to ensure that microbial replication during transit is controlled (22). These data are important considerations when endoscope samples are transported to off-site microbiology laboratories for culture.

One aspect of endoscope sample collection that is often overlooked when culturing endoscopes (3, 4, 7, 11, 17, 19, 23) is the need to use a “neutralizer” to ensure that trace residuals of antimicrobial agents (e.g., HLDs) are inactivated (9, 13, 15, 24). In addition, the use of a neutralizing agent ensures that organisms with sub-lethal injury are protected and stimulated to grow on culture thereby reducing VBNC issues when culture is used to determine if endoscopes are contaminated (9, 25). The neutralizer can be part of the extraction fluid (18) or it may be added immediately after sample collection (9, 13). In our study, neutralizer was added immediately after sample collection except for samples extracted using Letheen and Dey–Engley as these broths contain neutralizer. The efficacy of the neutralizer used has been demonstrated previously (26).

Despite using five replicates segments of PTFE-BBF segments (each segment was 5 cm), addition of neutralizer and concentration by centrifugation, there was wide variability in the detectable CFU (i.e., large SDs) of positive controls as well as samples collected from the PTFE-BBF channels with any of the extraction fluids tested. This reflects the variability of surviving culturable bacteria per cm^2^ in PTFE-BBF model as originally reported (13). As suggested by Neves et al. (5), use of longer PTFE segments for experiments may increase the level of culturable bacteria and thereby show less variability in viable counts. These data using the PTFE-BBF model underscore why culture of endoscopes that contain repeated rounds of glutaraldehyde-fixed residues may not be a reliable indicator of contamination even when optimal extraction fluid and friction are used. Despite very low CFU on culture, borescope examination showed there was a substantial accumulation of residual material in each of the 5 cm PTFE-BBF segments before sample collection. In addition, the borescope assessment supports the initial data reported by Alfa and Olson (13) confirming that the use of friction (i.e., bristle brush or pull-through device) for sample collection of the channel is critical to ensure optimal removal of fixed residuals—regardless of what fluid is used for sample extraction. Our data showed that the use of a tensioactive agent such as Tween80 in sample collection fluid was not sufficient to extract BBF material if the fluid was only flushed down the channel. Aumeran et al. (3) tested PBS, sterile water, and Letheen broth to evaluate which would be the most effective endoscope sample fluid. They reported that tensioactive agents in endoscope sample collection improved counts from biofilm and patient-used endoscope samples. However, the fluid with the tensioactive component that they evaluated was Letheen broth (which contains Tween80 as well as other neutralizing components such as lechithin). As shown by our data part of the improved recovery reported by Aumeran et al. (3) may be related to replication of bacteria in the broth rather than the impact of the tensioactive component of Letheen broth improving extraction efficacy. Alternatively, it may have been that this was the only fluid they evaluated that had neutralizing capability. Further studies are needed to determine any potential role of the tensioactive agent in terms of sample extraction efficiency from endoscope channels. From our testing using the PTFE-BBF model, there were no significant differences in the CFU recovered for RO-FBF versus Dey–Engley-FBF or Letheen-FBF when the transit times for all testing were less than 2 h at RT.

The pull-through channel cleaning device was the most effective at removing fixed residuals as visualized using the borescope (regardless of extraction fluid). This supports the conclusion by Cattoir et al. (11) regarding the efficacy of pull-through sample extraction and extends their findings as our PTFE data evaluated the glutaraldehyde-fixed BBF [not unfixed biofilm as used by Cattoir et al. (11)] as well as detection of low levels of bacteria (range 0.33–2.25 Log10 CFU/segment) using sample concentration for culture. Cattoir et al. (11) did not provide CFU data for their biofilm or non-biofilm PTFE channel model (they reported % recovery), so no comparison can be made regarding the efficacy of their recommended sample extraction protocol when low levels of bacteria are present. Detection of low levels of bacteria is an important consideration as Cattoir et al.’s (11) data on patient-used endoscopes confirms the low level of CFU detected (range of 1–158 CFU/endoscope) using their optimal sample collection protocol.

Our SEM results showed that the bristle brush left “tracks” of residual material that were similar to those observed by Ofstead et al.’s (19) borescope examination of the suction channel of reprocessed patient-used endoscopes. This variable surface contact of bristles may be a root cause explanation for how accumulation progressively gets worse in patient-used endoscopes that are repeatedly reprocessed when bristle brushes were used for cleaning (19, 27). This raises significant concerns for channels that cannot be brushed not only in terms of sample collection for culture but also in terms of cleaning of endoscope channels that may have BBF. Despite the pull-through being the most effective at removing BBF, it is not the ideal method for channel sample collection because there is significantly more aerosolization of material when the disks “pop” upon exit of the channel compared to when a bristle brush is the mechanism of friction. This results in loss of the channel sample and exposure of staff to biological material. Cattoir et al. (11) did not comment on this aspect. Aerosolization is a consideration for endoscope sample extraction; however, it is important to clarify that aerosolization does not occur when a bristle brush or pull-through device are used for manual cleaning as the endoscope is fully immersed in detergent during cleaning thereby eliminating aerosol generation.

The comparison of the efficacy of the CDC flush method to the FBF method using RO water as the extraction fluid in duodenoscope channels showed that *E. faecalis* was reliably extracted using the flush only method and that friction had little added advantage. Our data are similar to that of Cattoir et al. (11) who reported that for non-biofilm soiled PTFE recovery was optimal using saline or NPD flush-only extraction methods. However, the recovery of *E. coli* was significantly improved when friction was used for the cavity (*p* = 0.017). This suggests that the adhesion strength of Gram positives to the PTFE channel surface is different from that of Gram negatives before fixation. In addition, it is important to recognize that the unfixed material used in endoscope testing is easier to extract compared to fixed BBF. Indeed, the borescope examination of the PTFE-BBF channel post-sample collection showed that all methods that incorporated friction left far less residual material inside.

The lever cavity in duodenoscopes presents unique challenges to sample collection as there are moving parts as well as many small crevices that are difficult to adequately access. Recovery of both *E. faecalis* and *E. coli* from the lever cavity improved using FBF in both the 140 and 180 duodenoscopes, although the difference was statistically significant only for *E. coli*. This may reflect the improved loosening of material under the lever by the tiny bristle brush and improved collection due to the repeated “up-down” flushing of extraction fluid in the lever cavity collection protocol. Our findings support Gazdik et al.’s (4) report where a smaller flocked swab improved recovery of material from the cavity area compared to the CDC method using a very large bristle brush that did not fit into the smaller crevices of the lever cavity. Although we did not use fluorescent marker in our assessment, the recoverable CFU from duodenoscope lever cavity was shown to be significantly better when friction was used. Paula et al. (28) did use Gazdik et al.’s (4) sample collection method and found no viable organisms in 237 lever cavity samples tested. However, only the flocked swab was used to collect the lever samples and there was no flushing of the lever cavity and no neutralizer used during sample collection, so these factors may explain why there were no recoverable microorganisms. If desired the FBF lever cavity sample can be collected separately from the channel FBF sample using our collection protocols if site-specific samples are needed to further elucidate where the contamination is located.

In summary, we would recommend the use of RO water combined with friction using an appropriately sized sterile bristle brush for sample collection from endoscope channels and lever cavities of duodenoscopes. This method ensures compatibility with endoscope materials and fits best with sample collection in an endoscopy clinic as it does not require that the endoscope be reprocessed to remove collection fluid residuals. The endoscope can be used immediately after sample collection for a patient procedure or flushed with alcohol and dried if it is to be returned to storage. We would also recommend that a neutralizer be added immediately after sample collection and that during specimen transport the sample be held on ice. Finally, the sample collected should be concentrated by centrifugation or filtration (preferred) to ensure optimal sensitivity.

## Author Contributions

MA conceived of the research idea, cowrote the grant application, analyzed the data, and wrote the manuscript. HS conceived the research idea, cowrote the grant application, critiqued the experimental protocol, analyzed the data, and edited the manuscript. DD, GS, and CR critiqued the experimental protocol, analyzed the data, and edited the manuscript. PD and NO performed the experimental work, wrote and critiqued the experimental protocol, collated and analyzed the data, and edited the manuscript.

## Conflict of Interest Statement

MA is a consultant for Olympus, Novaflux, Johnson & Johnson, STERIS, 3M, Ofstead Associates. MA is on the advisory board for 3M and Johnson & Johnson. MA received research funds from STERIS, and 3M. HS is a member of the advisory board for Pendopharm and Ferring Canada and received research funds from Merck, Canada. DD is a member of the advisory board for Shire, Canada. GS, CR, PD, NO, and ZN have no conflict of interest to declare.
